# Transcriptome profiling of abdominal aortic tissues reveals alterations in mRNAs of Takayasu arteritis

**DOI:** 10.3389/fgene.2022.1036233

**Published:** 2022-11-16

**Authors:** Miao Yuqing, Gao Shang, Gao Qing, Wang Jiyang, Li Ruihao, Chen Zuoguan, Diao Yongpeng, Wu Zhiyuan, Li Yongjun

**Affiliations:** ^1^ Department of Vascular Surgery, Beijing Hospital, National Center of Gerontology, Institute of Geriatric Medicine, Chinese Academy of Medical Sciences, Beijing, China; ^2^ Graduate School of Peking Union Medical College, Chinese Academy of Medical Sciences, Beijing, China; ^3^ Department of Cardiovascular Surgery, Beijing Anzhen Hospital, Capital Medical University, Beijng, China

**Keywords:** Takayasu Arteritis (TA), interleukin, smooth muscle cell (SMC), bulk RNA sequencing, single cell RNA sequencing (scRNA)

## Abstract

Takayasu arteritis (TA) is a chronic granulomatous vasculitis involving in the main branches of aorta. Previous studies mainly used peripheral blood and some vascular tissues but seldom studies have sequenced vascular tissues. Here in this study, we aimed to explore the alterations of mRNA in TA by performing bulk RNA sequencing. A total of 14 abdominal aortic tissues including 8 from renal transplantation and 6 from patient with TA undergoing bypass surgeries. Bulk RNA sequencing were performed and after the quality control, a total of 1897 transcripts were observed to be significantly differently (*p* < 0.05 and Log_2_FC > 1) expressed between the TA and control group, among which 1,361 transcripts were in TA group and 536 in the Control group. Reactome Pathway Enrichment Comparison analysis revealed interleukin-10 signaling and signaling by interleukins were highly expressed in TA group. Besides, extracellular matrix organization was also observed in this group. WGCNA and PPI obtained 26 core genes which were highly correlated with the clinical phenotype. We then also perform deconvolution of the bulk RNA-seq data by using the scRNA-seq dataset and noticed the high proportion of smooth muscle cells in our dataset. Additionally, immunohistochemical staining confirmed our bioinformatic analysis that TA aortic tissues express high levels of IL-1R1 and IL-1R2. Briefly, this study revealed critical roles of interleukins in TA pathogenesis, and SMCs may also participate in the reconstruction in vessel wall at late stage of TA.

## Introduction

As a chronic inflammatory disease of large vessels, Takayasu Arteritis (TA) is characterized by granulomatous inflammation, primarily affecting the aorta and its branches (Kerr et al., 1994). Present treatment for TA is mainly based on glucocorticoids and immunotherapy without specific drugs, but more than 30% of patients eventually experienced vascular complications requiring surgical interventions (Comarmond et al., 2017). Our previous study has demonstrated the efficacy and safety of surgical management for TA, including open surgery and endovascular repair (Chen et al., 2018; Diao et al., 2020; Miao et al., 2020). However, it remains a major clinical issue to deal with the restenosis of culprit vessels due to inflammation progression.

Furthermore, to construct effective animal model and further research, studies on the pathogenesis of TA is necessary. Human specimens were then collected and adopted for TA-related research but most published studies focused on peripheral blood including plasma, serum and blood cells. Our team has explored the cellular heterogeneities of peripheral blood and found that CD14 monocytes and gene expressions involving in oxidative stress were increased in TA patients by applying single-cell RNA sequencing (scRNA-seq) (Qing et al., 2021). Profiled chemokines with peripheral blood, suggesting its important roles in TA (Kong et al., 2022). Except the two blood RNA profiling above, studies on RNA sequencing of TA tissues has been seldom reported.

Previous studies have revealed critical roles of monocyte and fibroblast in TA (Wei et al., 2015; Wu et al., 2021a; Qing et al., 2021), and some studies have shown that interleukin (ILs) play a critical role in TA, especially IL-6 (Kong et al., 2016; Yang et al., 2016; Choy et al., 2020; Ma et al., 2020; Kadoba et al., 2021). A previous CANTOS study has shown the IL-1β monoclonal antibody, Canakinumab, may play an important role in atherosclerosis (Ridker et al., 2017). Increased expression of IL-1β and IL-1R2 genes in peripheral blood mononuclear cells (PBMC) of patients with TA can be induced by Toll-Like receptor 4 activation (Kabeerdoss et al., 2022), but the role of IL-1β in TA remains unknown. Furthermore, none of these studies used vascular tissues for RNA sequencing, and the overall changes in TA at the transcriptome level are unknown. So, we performed bulk-RNA sequencing by using abdominal aorta samples from both TA patients and donors. At the same time, the scRNA-seq technology was used to initially explore cellular types in the vascular tissue of TA patients, which is adopted as a reference to perform a deconvolution analysis on the bulk RNA-seq data. Lastly, we explored the roles of ILs in TA.

## Method and materials

### Patients and ethic approval

This study enrolled eight TA patients and eight kidney transplant donors as controls between August 2018 and December 2021 at Beijing Hospital in Beijing, China. TA was diagnosed according to the criteria of the American College of Rheumatology (Arend et al., 1990) and all patients underwent surgical revascularization. All donors of kidney transplantation information were kept confidential. Bulk RNA sequencing was performed in six patients and all the donors. Single-cell RNA sequencing was explored in the other two patients with TA.

The study protocol was approved by the Ethics Committees of Beijing Hospital (2018BJYYEC-030-01). Written informed consent was obtained from all the patients in this study. Human samples were used only in experiments in compliance with applicable regulations.

### Total RNA extraction

According to manual instructions, total RNA was extracted from tissues using Trizol (Invitrogen, Carlsbad, CA, United States). Approximately 60 mg of tissues were ground into powder by liquid nitrogen in a 2 ml tube, then homogenized for 2 min and rested horizontally for 5 min. The mix was centrifuged for 5 min at 12,000 × g at 4°C, then the supernatant was transferred into a new EP tube with 0.3 ml chloroform/isoamyl alcohol (24:1). Mixture was shaken vigorously for 15 s, then centrifuged at 12,000 × g for 10 min at 4°C. Then, transfer the supernatant to the 1.5 ml centrifuge tube, add 2/3 volume of isopropyl alcohol. Gently invert and mix, place in −20°C refrigerator for more than 2 h. After deserting the supernatant, the RNA pellet was washed twice with 1 ml 75% ethanol, then the mix was centrifuged at 13,600 rpm for 3 min at 4°C to collect residual ethanol, followed by the pellet air dry for 5–10 min in the biosafety cabinet. Finally, 25 µL–100 µL of DEPC-treated water was added to dissolve the RNA. Subsequently, an Agilent 2100 bioanalyzer (Thermo Fisher Scientific, MA, United States) was used to quantify and qualify the total RNA.

### mRNA library construction

DNase I was used to digest double-stranded and single-stranded DNA in total RNA, then, transfer 75 μL magnetic beads into reaction products of DNase I and Incubate at room temperature for 5 min for purification. RNase H or Ribo-Zero method (human, mouse, plants) (Illumina, United States) was used to remove the rRNA. Using fragment buffer at the appropriate temperature, purified mRNA was fragmented into small pieces. Then, First-strand cDNA was generated in First Strand Reaction System (MGIEasy DNA Adapters-16, 1000005284) by PCR, and the second-strand cDNA was generated as well. The reaction product was purified by magnetic beads, afterwards, A-Tailing Mix and RNA Index Adapters were added by incubating to carry out end repair. PCR was used to amplify cD NA fragments with adapters, and Ampure XP beads were used to purify the products. Library was validating on the Agilent Technologies 2100 bioanalyzer for quality control. With the splint oligo sequence, the double stranded PCR products above were heated denatured and circularized. Final library formatted from single strand circular DNA (ssCir DNA). The final library was amplified with phi29 (Thermo Fisher Scientific, MA, United States) to make DNA nanoball (DNB) which had more than 300 copies of one nanoball, DNBs were loaded into the patterned nanoarray, and single end 50 bases reads were generated on BGISEQ500 platform (BGIShenzhen, China).

### Single-cell suspensions preparation

Renal artery specimens were collected from TA patients and digested in two different enzymes, including a GEXSCOPE kit (Singleron Biotechnologies) and a self-made enzyme cocktail, which is pending patent application. The self-made enzyme cocktail was consisted with Collagenase A (Roche, 10103578001), Collagenase II (Gibco, 17101-015), Hyaluronidase I (Sigma, H3506), DNase I (Sigma, D4527), DMEM/F-12 medium (STEMCELL, 36254) and Fetal Bovine Serum (GEMINI, 900-108). Single-cell suspension samples were loaded onto the microfluidic device and scRNA-seq libraries were constructed by the GEXSCOPETM Single-Cell RNA Library Kit (Singleron Biotechnologies) according to the Singleron GEXSCOPETM protocol. The sequencing range is about 150 bp in the tail.

### Differential genes and pathway analysis

Regarding the bulk RNA-seq analysis, differentially expressed genes (DEGs) between two groups were obtained with default parameters. Significant DEGs were set as *p*-value ≤ 1e−05. Gene Ontology (GO), Kyoto Encyclopedia of Genes and Genomes (KEGG) pathway, Reactome (Yu and He, 2016; Wu et al., 2021b) were applied to perform the functional pathway annotation.

### Single cell RNA sequencing

The protocol for single-cell RNA sequencing followed the instructions of the GEXSCOPETM Single-Cell RNA Library Kit (Singleron Biotechnologies). Details have been described and can be referred to Gao et al. (Qing et al., 2021).

### Weighted gene coexpression network analysis

We calculated the MAD (medium absolute deviation) of each gene separately, removed the top 50% genes with the smallest MAD. Then, we removed the outlying genes and samples by using the good samples genes method of WGCNA, one of R software packages, and further constructed the scale-free co-expression-network using WGCNA. Specifically, at first, the Pearson’s correlation matrices and average linkage method were both performed for all pair wise genes, then, a weighted adjacency matrix was constructed using a power function A_mn = |C_mn|^ β (C_mn = Pearson’s correlation between Gene_m and Gene_n; A_mn = adjacency between Gene m and Gene n). β was a soft-thresholding parameter that could emphasize strong correlations between Genes and penalize weak correlations. After that, the adjacency was transformed into a topological overlap matrix (TOM), which could measure the network connectivity of a Gene defined as the sum of its adjacency with all other Genes for network Gene ration, and the corresponding dissimilarity (1-TOM) was calculated. To classify Genes with similar expression profiles into Gene modules, average linkage hierarchical clustering was conducted according to the TOM-based dissimilarity measure with a minimum size (Gene group) of 30 for the Genes dendrogram. Set the sensitivity as: 3, to further analyze the module, we calculated the diversity of module genes, choose a cut line for module dendrogram, and merged some modules. In addition, we also merged modules with a distance of less than 0.25. Screening for hub genes, we calculated the correlation between module feature vector and gene expression to obtain MM, and all significant modules were selected as hub genes.

### Construction and analysis of protein interaction network

String database (http://string-db.org/) aims to collect, score, and integrate all publicly available sources of protein-protein interaction information and to supplement these sources by computational predictions. Cytoscape can provide researchers with biological network analysis and two-dimensional (2D) visualization. In this study, the string database was used to construct a PPI network for predicting key genes (confidence >0.4). We took the intersection of hub genes related to clinical phenotypes which were output from WGCNA and DEGs, then imported the intersection data into the string website to find the correlation between gene expression proteins, and then processed it with Cytoscape to search for the predicted hub genes. The hub genes are found in the core module through MCODE, and then the top ten are calculated by two algorithms (MCC and MNC) and exported after visualization.

### Deconvolution

To estimate the cell-type specific proportions in the bulk-seq dataset, deconvolution was performed by using MuSic (27). Regarding the deconvolution by MuSic. The scRNA-seq dataset was set as reference for the downstream analysis. Default parameters were adopted for the deconvolution analysis.

### Addmodule scores

AddModuleScore, was applied to calculate module score in each cell. Candidate genes for the interleukin were finally listed in Supplementary Table S1.

### Immunohistochemistry

To evaluate the expression of ILs in vascular tissue, immunohistochemistry for IL1R1 (ab106278, Abcam, MA, United States), IL1R2 (ab212208, Abcam, MA, United States) and IL6 (ab290735, Abcam, MA, United States), SMA (ZM-0003, ZSGB-BIO, Beijing, China) staining was performed.

## Results

### Clinical characteristics of the included patients with Takayasu arteritis

**FIGURE 1:**
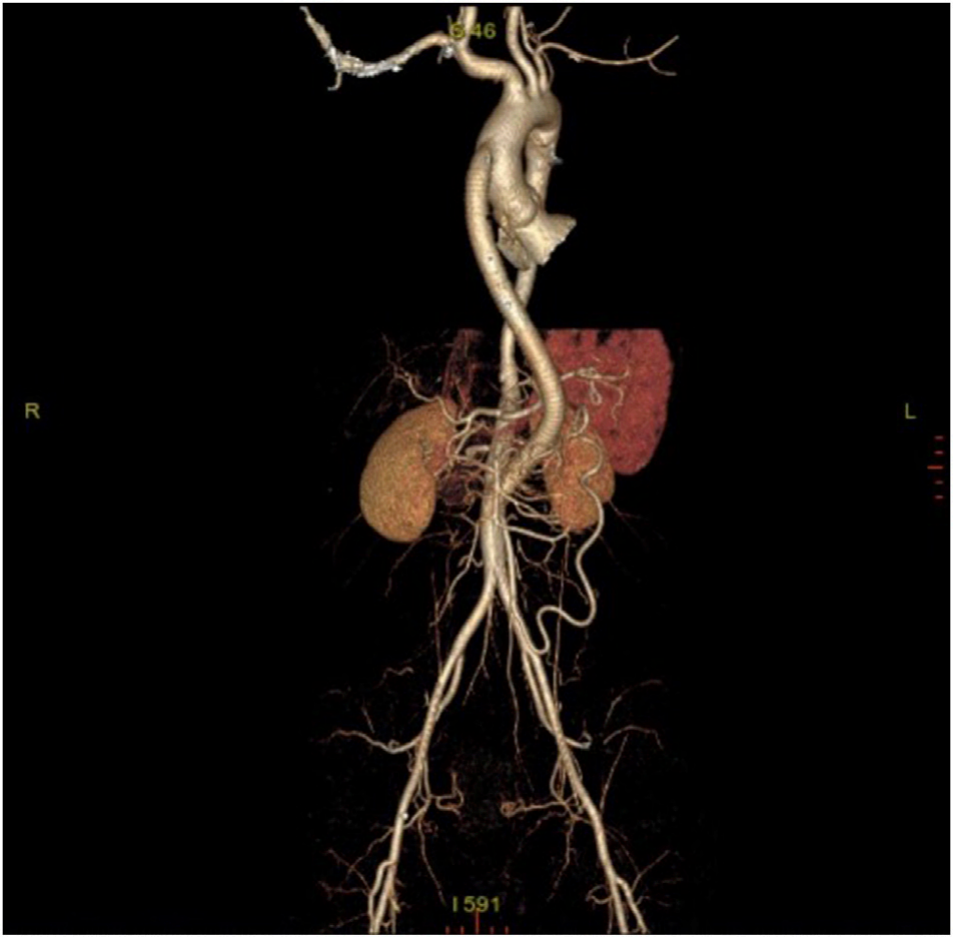
Post-operative computerized tomographic angiography (CTA) of patient with TA.

A total of 118 patients was admitted to Beijing Hospital with TA between August 2016 and August 2021, 104 (88.1%) of which was female. The mean age was 31.68 ± 12.98 years old. In this study, bulk RNA sequencing was performed for six patients with TA underwent surgical revascularization, whose clinical characteristics are detailed in Table 1. Here it is a case underwent bypass for ascending aorta-abdominal aorta. Figure 1 is the postoperative CTA.

**TABLE 1:** Clinical Characteristics of TA patients.

Case no	TA1	TA2	TA3	TA4	TA5	TA6
Gender	Female	Female	Female	Female	Female	Male
Age (years)	22	28	44	54	26	16
Course of disease (years)	0.25	6	20	30	0.7	3
Blood pressure (mmHg)	125/87	170/81	116/74	118/96	148/72	132/77
Dizziness	None	None	9 years	None	None	3 years
Blurred vision	None	None	None	None	None	None
Temperature (°C)	36.2	36.4	36.2	36.3	36.4	36.3
ESR(mm/hr)	2	3	10	23	8	1
CRP	0.2	0.5	0.3	0.4	0.1	<0.1
Vascular pain in the past 3 months	None	None	None	None	None	None
NIH class	Inactive	Inactive	Inactive	Inactive	Inactive	Inactive

ESR, erythrocyte sedimentation rate; CRP, C-reactive protein; NIH, National Institutes of Health.

### Altered transcriptome profiling revealed by bulk RNA sequencing in abdominal aortic tissues of patients with Takayasu arteritis

**FIGURE 2:**
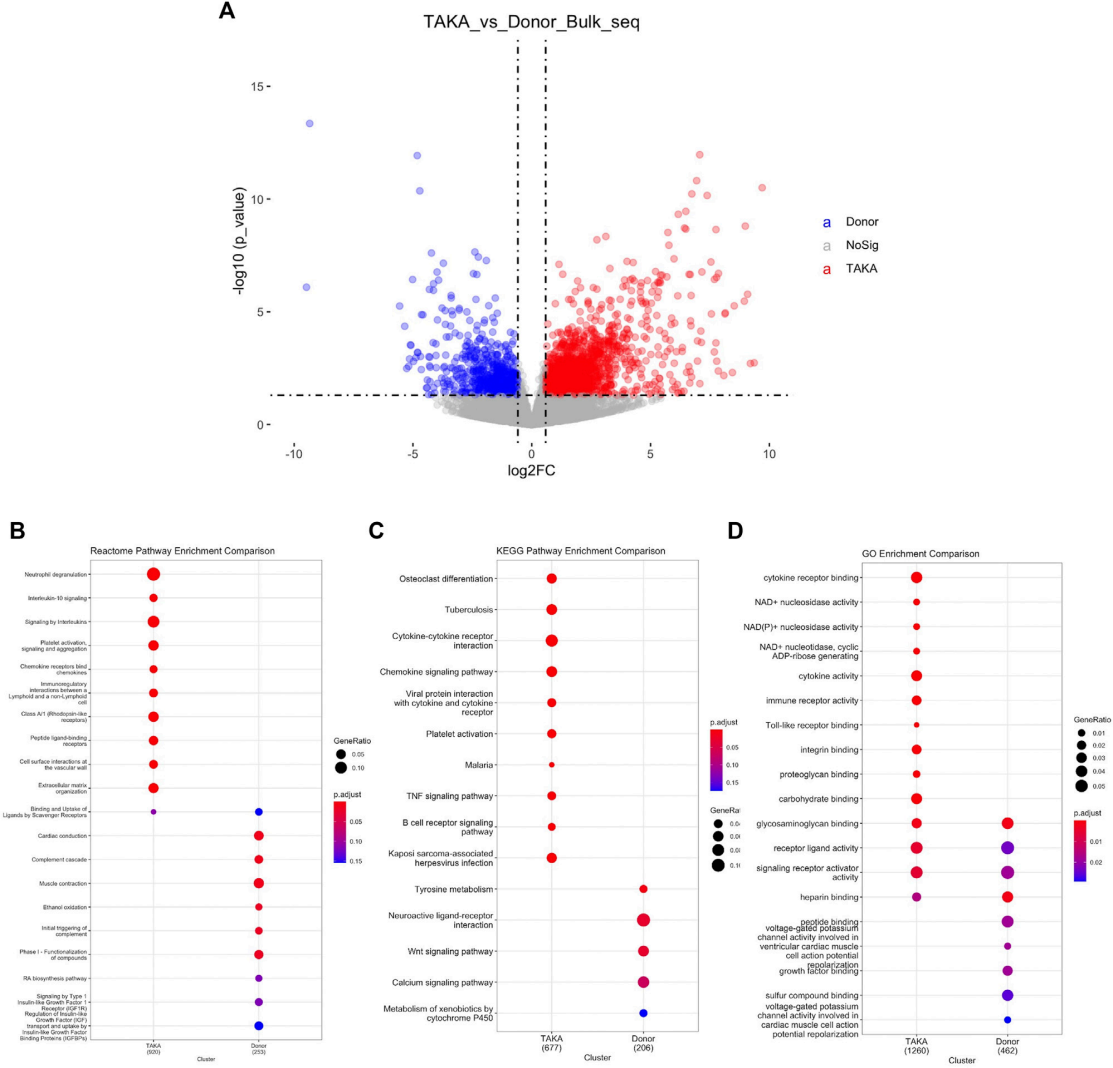
Analysis of differential expressed genes. **(A)** Volcano plot displaying differential expressed genes. 1897 transcripts were significantly differently (*p* < 1e-05 and Log_2_FC > 1) expressed between the TA and donor group. Pathway analysis was perfomed in **(B)** Reactome pathway comparison **(C)** KEGG pathway enrichment comparison and **(D)** GO enrichment comparison.

To explore the transcriptome profiling of abdominal aortic tissue in patients with TA, bulk RNA-Seq was conducted by using specimen from six patients with TA and eight donors. A total of 18977 genes were detected with an average alignment rate of 94.2%. After the quality control, a total of 1897 transcripts were observed to be significantly differently (*p* < 0.05 and Log_2_FC > 1) expressed between the TA and donor group, among which 1,361 transcripts were in TA group and 536 in the donor group, as shown in Figure 2A.

To identify possible biological alterations related to TA, gene ontology (GO) and pathway analyses were conducted. Two programs (KEGG pathway, Reactome) were used for pathway enrichment analysis. The Reactome pathway enrichment analysis resulted in enriched interleukin-related signaling such as interleukin-10 signaling, signaling by interleukins in TA group (Figure 2B). KEGG pathways enrichment also revealed in upregulated signaling like cytokine-cytokine receptor interaction, chemokine signaling pathway in TA group (Figure 2C). The top ten items from GO were selected based on the enrichment score. The most significant biological processes related to Cytokine receptor binding, Cytokine activity, Immune receptor activity (Figure 2D).

### WGCNA and PPI analysis for bulk RNA-seq datasets

**FIGURE 3:**
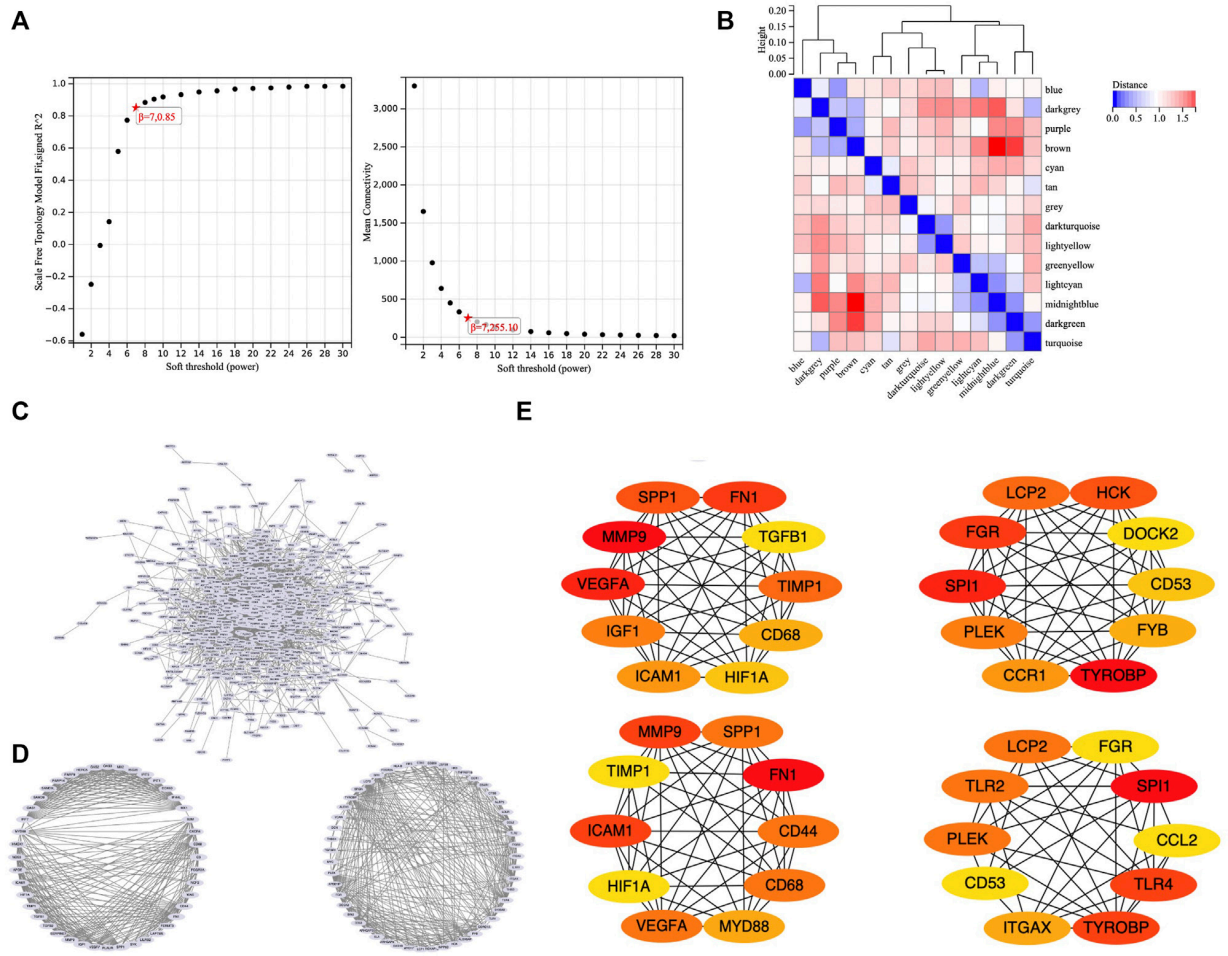
WGCNA and PPI. **(A)** Soft threshold power was set to 7. **(B)** Hierarchical clustering tree was constructed for all genes, and 14 important modules were identified. **(C)** A total of 687 genes were extracted from hub genes extracted from WGCNA and 1806 differentially expressed genes. **(D)** Two core modules screened out by the MCODE plug-in. **(E)** 26 core genes identified by MCC algorithm and MNC algorithm.

To obtain an optimal soft threshold power in weighted gene co-expression network analysis (WGCNA), a network topology analysis was performed and was finally set at 7 (Figure 3A). To classify genes with similar expression profiles into gene modules, average linkage hierarchical clustering was conducted according to the TOM-based dissimilarity measure with a minimum size (Gene group) of 30 for the Genes dendrogram. Hierarchical clustering trees of all genes were constructed, and 14 important modules were generated (Figure 3B). The dendrogram and Heatmap of genes showed no significant differences in the interactions between different modules, indicating a high degree of independence between these modules (Supplementary Figure S1). The tan, blue, purple, turquoise, lightyellow, cyan module had the negative correlation with the status of TA (Supplementary Figure S2). We calculated the expression correlation between the module feature vector and the gene to obtain the module membership (mm). Taking the MM cut-off value of 0.8 (|mm|>0.8) as the measurement standard, a total of 5,094 genes were selected as differentially expressed genes due to their high correlation with the clinical phenotype.

To set up the construction and perform the analysis of protein-protein interaction (PPI) network, we collected the hub genes extracted from WGCNA and 1806 differentially expressed genes, finally obtained 687 genes (Figure 3C). Our PPI network was based on these 687 genes, constructed by the string online database, and analyzed by Cytoscape software. We screened out two core modules using the MCODE plug-in (Figure 3D) and identified hub genes using MCC algorithm and MNC algorithm respectively (Figure 3E). Finally, a total of 26 core genes were obtained. The relationship of 26 hub genes to structural cells and immune cells was analyzed. (Supplementary Figure S3). We can learn that some hub genes were mainly expressed in structure cells like, *CCL2, ICAM1, IGF1, VEGFA*; others are mainly expressed in immune cells like *CCR1, CD53, CD68, DOCK2, FYB1, HCK, ITGAX, LCP2, MYD88, PLEK, SPI1, SPP1, TGFB1, TLR2, TLR4, TYROBP*. Besides, some genes were expressed in both types like *CD44, FGR, FN1, HIF1A, TIMP1*.

### Deconvolution of bulk RNA-seq dataset revealed high proportion of structure cells in abdominal aortic tissues in Takayasu arteritis patients

**FIGURE 4:**
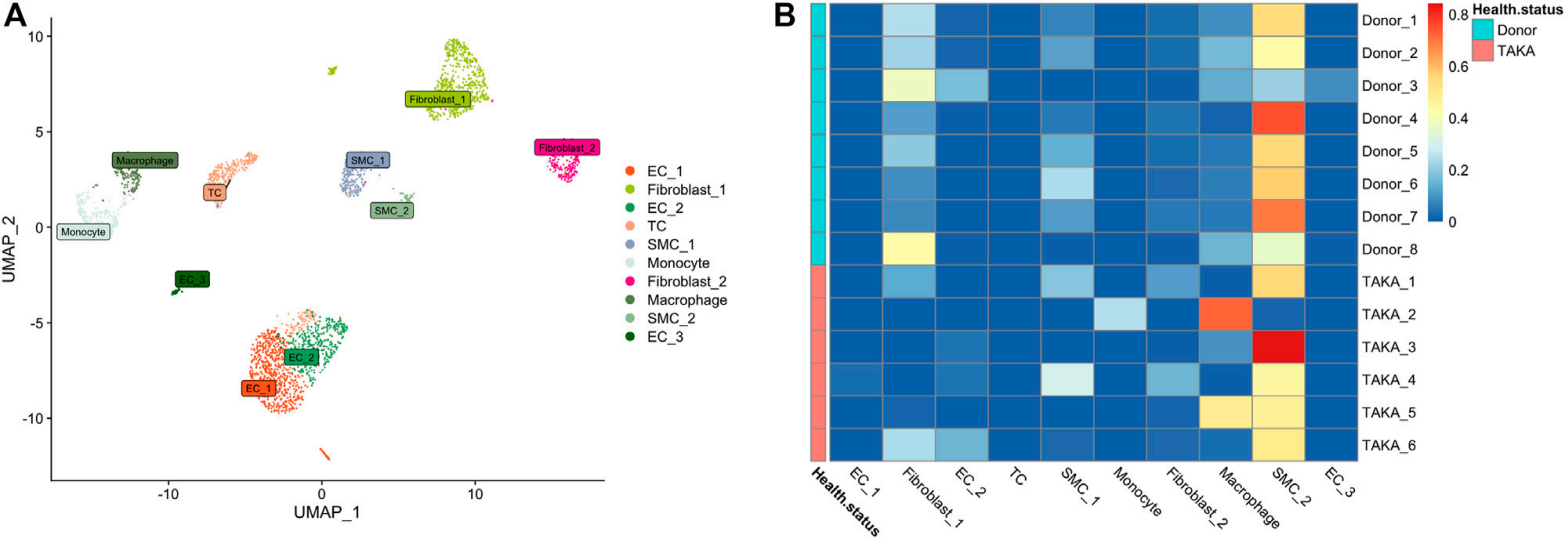
**(A)** 10 cell types **(B)** Deconvolution analysis was performed using cluster specific markers identified in scRNA-seq datasets. Several types of cells make up vascular tissue, including SMCs, fibroblasts, and macrophages. EC = endothelial cell, TC = T cell, SMC = smooth muscle cells.

Previously, we explored the cellular heterogeneities of vascular tissues in TA patients and harvested a total of 2920 cells for downstream analysis. Unbiased clustering revealed 10 cell types, including three types of endothelial cells, two types of vascular smooth muscle cells (SMCs) and fibroblasts, T cells, monocyte, and macrophages (Figure 4A; Supplementary Figures S4, S5).

To estimate the cell-type specific proportions in the bulk-seq dataset, deconvolution was performed by using MuSic (27). The cluster specific markers identified in scRNA-seq dataset were taken as reference for the deconvolution analysis with default markers. Finally, as shown in Figure 4B, the main cell types in vascular tissue were SMCs, fibroblasts, and macrophages.

### Interleukins may play a critical role in the pathogenesis of Takayasu arteritis

**FIGURE 5:**
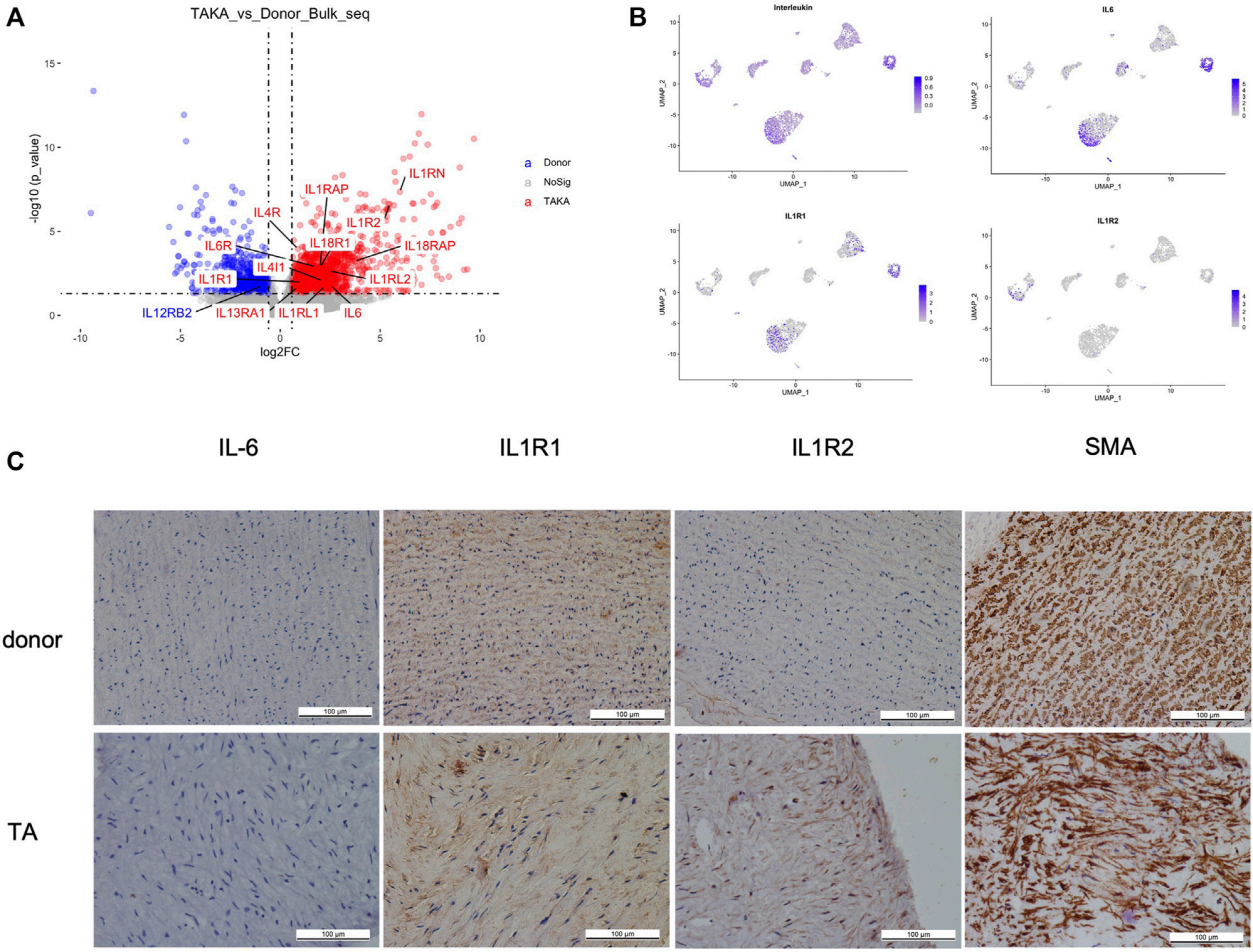
**(A)** expression of interleukins **(B)** cell types of interleukins expression **(C)** IHC staining of IL6, IL1R1, IL1R2 and SMA in patients with TA and donors.

As indicated in previous analysis, interleukins showed high signaling in TA tissues. We then explored the expression of interleukins in both bulk-seq and scRNA-seq datasets. As shown in Figure 5A, a list of interleukins was identified to be highly expressed in TA tissues such as IL-6, IL1R1, IL1R2. We then performed AddModuleScore analysis on interleukins in the scRNA-seq datasets and noticed that they were mainly expressed in EC1, fibroblast 2 and monocytes. Specifically, IL-6 mainly presented in EC1&fibroblast2, IL1R1 in EC1& fibroblast2 while IL1R2 in monocytes (Figure 5B). Then we performed IHC staining of these ILs as shown in (Figure 5C), corresponding to the *in silico* analysis.

## Discussion

Mechanisms and pathogenesis of TA have been studied for a time but many issues remain unclear including the pathogenesis, the mechanism of downstream effectors, role of vessel structural cells and well-established animal model. Current theories of TA pathogenesis have been linked to adventitia origin, infiltration of inflammatory cells in vascular media, and activation of inflammatory mediators (Tombetti and Mason, 2019; Watanabe et al., 2020a; Watanabe et al., 2020b).

To the best of our knowledge, this study firstly performed bulk RNA-sequencing of TA vessel tissues to obtain the overall alterations of transcriptome in TA. Regarding the materials used for TA research, most studies detected RNA, cytokines, chemokines by using peripheral blood samples (Matsumoto et al., 2021; Qing et al., 2021; Kabeerdoss et al., 2022; Kong et al., 2022). In this way, several susceptibility locus have been identified by genome-wide association studies to pinpoint potential clues for new therapeutic targets (Renauer et al., 2015; Ortiz-Fernández et al., 2021). However, due to scarce vessel specimen obtained from the open surgical repair, studies in TA with tissues are still limited.

Pathway enrichment analyses, both Reactome and KEGG pathway, revealed that cytokines and chemokines are playing a critical role in the pathogenesis of TA. Evaluated chemokines in vessel tissues of TA with immunofluorescence and immunohistochemistry staining and identified CCL22, RANTES, CXCL16, CXCL11, and IL-16 as the major chemokines recruiting immune cells in the vascular tissue of patients with TA (Kong et al., 2022). As to cytokines and its receptor, found out increased expression of *IL-1R2* genes in PBMCs of patients with TA. Similarly, in this study, *IL1R* was highly expressed in TA (Kabeerdoss et al., 2019). *IL1R1* mainly presented in EC1& fibroblast2 while *IL1R2* in monocytes. The results of *IL1R1* and *IL1R2* IHC staining confirmed these bioinformatic analysis. Examined the whole blood RNA and draw a conclusion that IL-1 signaling pathway related to the advanced stage of disease and a poor prognosis (Matsumoto et al., 2021). IL-1 and IL1R may be a potential target of TA treatment, in which further studies are required. Interestingly, in the KEGG pathway enrichment analysis, *tuberculosis* (TB) is upregulated in TA, providing new evidence of the association of TA and TB. Active TB is more prevalent in the TA population than in the general population (Lim et al., 2016). While in some studies, TB was considered as a potential trigger for TA (Soto et al., 2012).

Weighted gene coexpression network analysis is an analysis method for analyzing gene expression patterns of multiple samples. It can cluster genes with similar expression patterns and analyze the correlation between modules and specific traits or phenotypes. It is widely used in the research of phenotypic traits and gene association analysis. WGCNA revealed that a total of 5,094 genes corelated with clinical phonotype. While in the PPI network, we obtained 26 core genes. According to gene function, core genes are divided into 2 categories, immune response, and angiogenesis. Nineteen genes related to immune response, such as *SPP1, TGFB1, CD68, CD44, ICAM1, MMP9, LCP2, HCK, DOCK2, CD53, FYB, TYROBP, CCR1, CCL2, ITGAX, MYD88, TLR2, TLR4 and SPI1*. The rest 7 genes associated to vessel remodeling, participated by SMCs, including *FN1, TIMP1, HIF1A, IGF1, VEGFA, PLEK AND FGR. TGFB1* encodes a secreted ligand of the TGF-beta (transforming growth factor-beta) superfamily of proteins, a profibrotic cytokine in M2 (Cui et al., 2020). Compared the TGF-β level in serum of TA patients and healthy control and drew a conclusion that TGF-β is lower in TA, especially active TA (Savioli et al., 2020). It demonstrated that TGF-β is a potential biomarker of disease activity assessment. TLR was a hotspot in the research of immune system disease. Revealed that TLR4 activation increased expression of IL-1β and IL-1R2 genes in PBMCs of patients with TA (Kabeerdoss et al., 2022). In this study, we found both TLR4 and IL1R2 highly expressed in tissues of patients with TA, on which further study is needed. Other genes were identified as biomarker of TA diagnosis or disease activity assessment including *TIMP1, ICAM1, VEGF, MMP-9* (Tripathy et al., 2008; Sun et al., 2012; Dogan et al., 2014; Machado et al., 2018; Cui et al., 2019).

Deconvolution estimated cell-type composition that SMCs accounted for the most cells, while TCs the least. The reason for this condition may lie in the vessel structural remodeling of patients needing surgical intervention. In the advanced stage of TA, phenotypic switching of SMC may be the key process of vessel stenosis. In this study, immunohistochemistry indicated disorganized SMCs. While rare study focused on the function of SMCs in TA. Further research is required. T lymphocytes play a key role in TA. Many kinds of T cells, such as CD4^+^ T cells, CD8^+^ T cells, γδT cells, activated in the procedure of TA. (Mirault et al., 2017).

Several limitations of this study need to note. Firstly, the sample size is limited, more samples are required for future studies. The clinical background of the renal transplantation is confidential, limiting our knowing of the gender and age of the donors. Besides, how the transcriptomes altered at different positions of the aorta and its branches remains unknown.

## Conclusion

Our bulk RNA-seq profiling of abdominal aortic tissues revealed high expression of immune-related activities and vascular remodeling in TA. IL1R1 and IL1R2 may play critical roles in pathogenesis of TA. Phenotypic switching of SMC may be a key process in the vascular remodeling of TA. But more robust studies are required.

## Data Availability

The data presented in the study are deposited in the GSA repository, accession number HRA003348.
