# Supplementary figures and images for: Transcriptome profiling of abdominal aortic tissues reveals alterations in mRNAs of Takayasu arteritis
Source: Front Genet. 2022 Nov 16;13:1036233. doi: 10.3389/fgene.2022.1036233 (PMC9709398; doi:10.3389/fgene.2022.1036233)

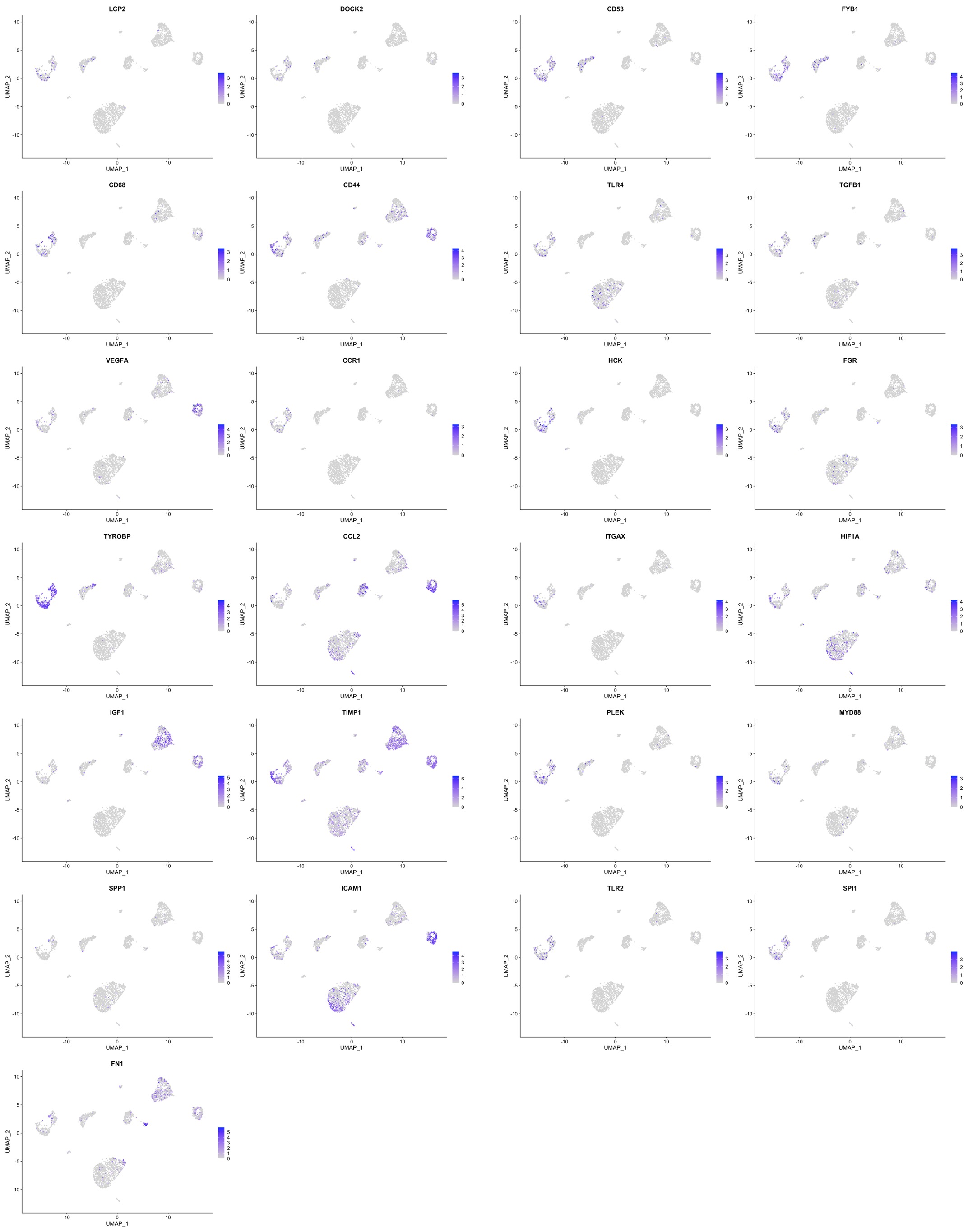

Supplement: Supplementary file 1 [file Image3.JPEG]

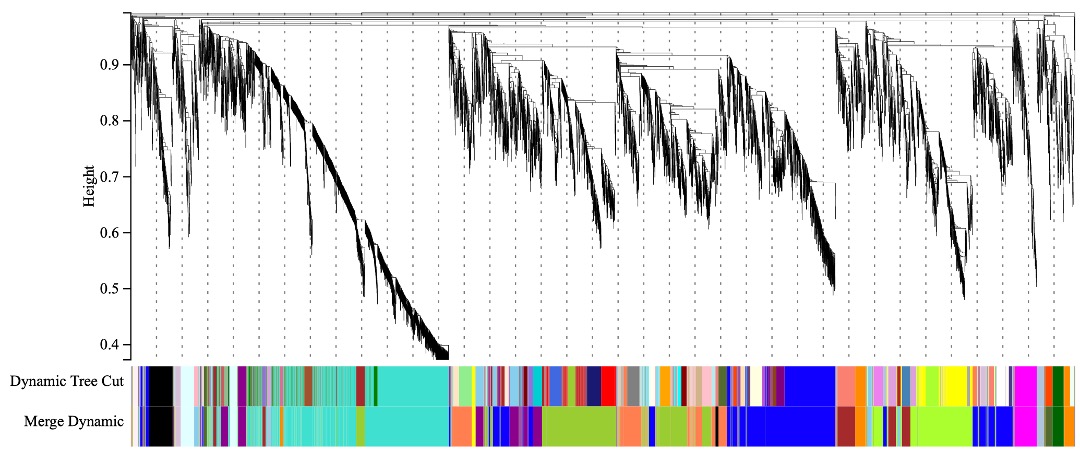

Supplement: Supplementary file 2 [file Image1.JPEG]

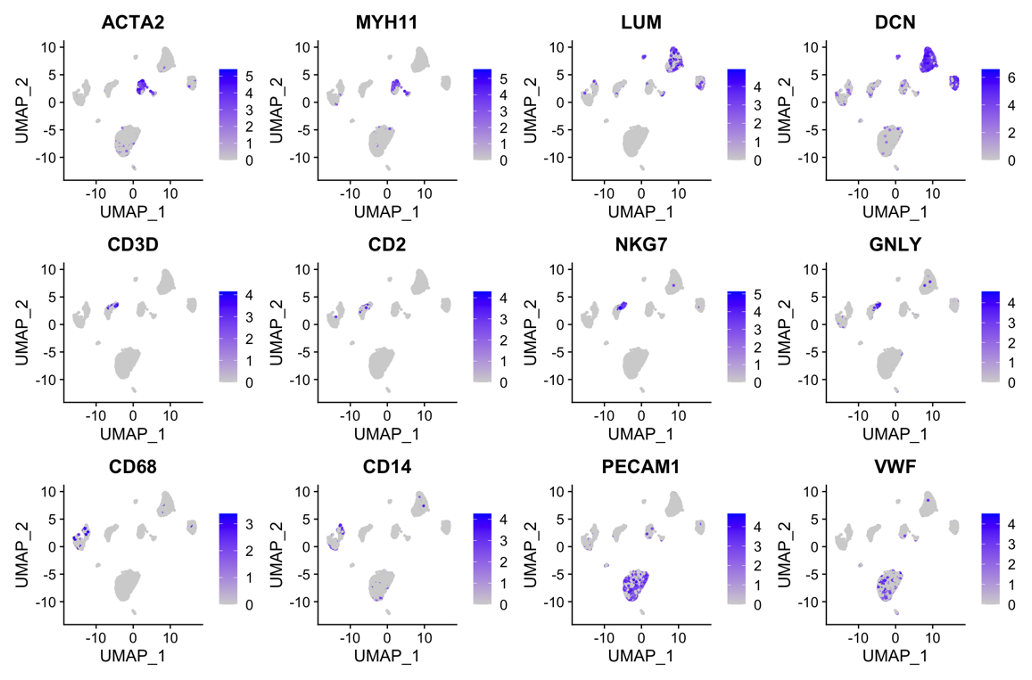

Supplement: Supplementary file 3 [file Image4.JPEG]

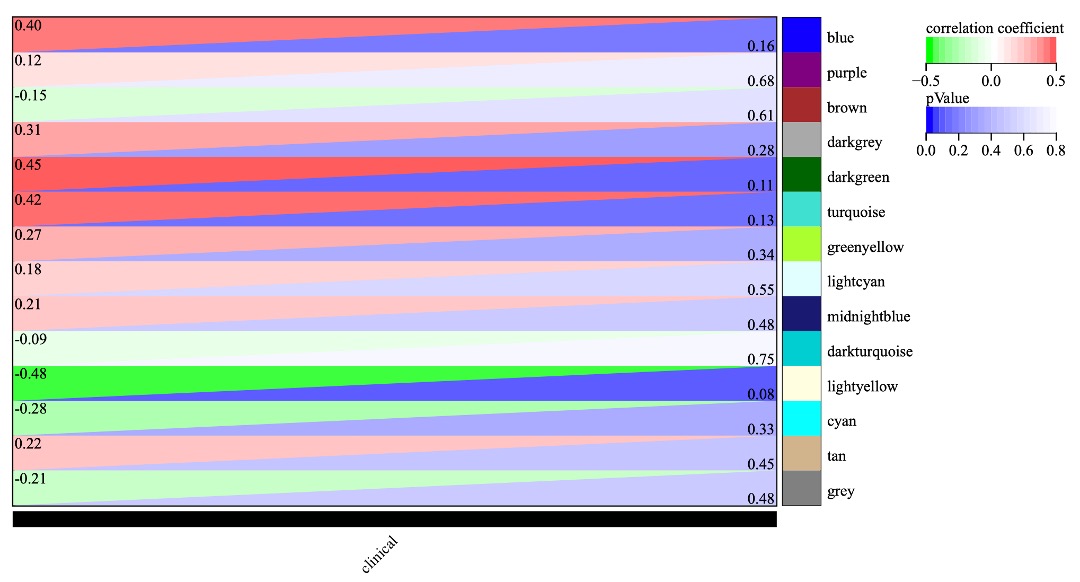

Supplement: Supplementary file 4 [file Image2.JPEG]

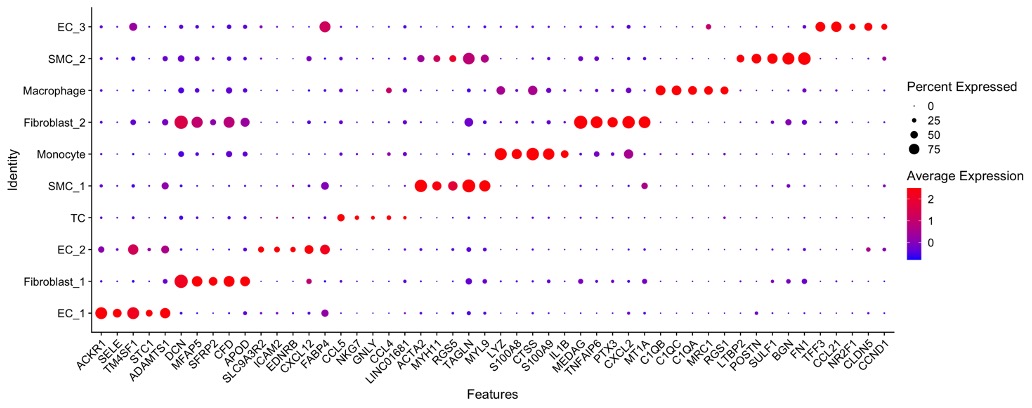

Supplement: Supplementary file 5 [file Image5.JPEG]
